# Engineered T Cells for the Adoptive Therapy of B-Cell Chronic Lymphocytic Leukaemia

**DOI:** 10.1155/2012/595060

**Published:** 2011-08-08

**Authors:** Philipp Koehler, Patrick Schmidt, Andreas A. Hombach, Michael Hallek, Hinrich Abken

**Affiliations:** Department I of Internal Medicine, and Center for Molecular Medicine Cologne, University Hospital Cologne, Robert-Koch-Strasse 21, 50931 Cologne, Germany

## Abstract

B-cell chronic lymphocytic leukaemia (B-CLL) remains an incurable disease due to the high risk of relapse, even after complete remission, raising the need to control and eliminate residual tumor cells in long term. Adoptive T cell therapy with genetically engineered specificity is thought to fulfil expectations, and clinical trials for the treatment of CLL are initiated. Cytolytic T cells from patients are redirected towards CLL cells by ex vivo engineering with a chimeric antigen receptor (CAR) which binds to CD19 on CLL cells through an antibody-derived domain and triggers T cell activation through CD3**ζ** upon tumor cell engagement. Redirected T cells thereby target CLL cells in an MHC-unrestricted fashion, secret proinflammatory cytokines, and eliminate CD19^+^ leukaemia cells with high efficiency. Cytolysis of autologous CLL cells by patient's engineered T cells is effective, however, accompanied by lasting elimination of healthy CD19^+^ B-cells. In this paper we discuss the potential of the strategy in the treatment of CLL, the currently ongoing trials, and the future challenges in the adoptive therapy with CAR-engineered T cells.

## 1. Introduction

B-cell chronic lymphocytic leukaemia (B-CLL) is the most common leukaemia in the western hemisphere with escalating incidence. Although treatment of B-CLL has achieved significant progress during the last years based on the use of nucleoside analoga, monoclonal antibodies, and bone marrow transplantation [1–5], the disease is rarely cured, even in those patients with complete molecular remission [6–8]. Interest is therefore growing in activating the immune system, by single agents or in combination with chemotherapy, to control the disease. The application of monoclonal antibodies, including anti-CD20 and anti-CD52 antibodies, substantially improved response rates and progression-free survival [9]. Allogeneic haematopoietic stem cell transplantation induced a significant T cell-mediated graft-versus-leukemia response and durable remissions in a subset of patients with chemotherapy-refractory B-CLL [4, 5]. Genetically modified malignant cells enhanced the antitumor response [10, 11]. The isolation of B-CLL-reactive T cells from patients with long-lasting tumor regression [12] sustained the concept that adoptive cell therapy with CLL specific T cells may be successful in controlling the disease. Advances in genetic engineering of a recombinant T cell receptor (TCR) and of a chimeric antigen receptor (CAR) provide the technology to modify T cells ex vivo with predefined specificity for use in specific cell therapy. This paper summarizes recent experiences with CAR-engineered T cells for the use in adoptive therapy of B-CLL.

## 2. **Redirecting** T Cells** towards B-CLL**

Tumor-specific T cells can be genetically engineered in large quantities by engrafting with a recombinant TCR or alternatively with a CAR of predefined tumor specificity. In contrast to the TCR, the CAR consists of one trans-membrane polypeptide chain; the extracellular domain is composed of a single chain fragment of variable region (scFv) antibody for binding; the intracellular domain provides T cell activation through the CD3*ζ* endodomain upon antigen engagement [13–15]. The “T-body” concept thereby combines the power of the targeting antibody with the effector mechanisms of cytolytic T cells [14, 16]. The CD3*ζ* molecule contains three immunoreceptor tyrosine-based activation motifs (ITAMs) which are phosphorylated to initiate T cell activation; the first and third ITAMs additionally cause apoptosis. Inactivation of these ITAMs by mutation consequently decreased apoptosis and enhanced survival of redirected T cells upon CAR signalling [17–19]. By using an antibody for target recognition, CAR-redirected T cells bypass the MHC haplotypes of the individual patients and undergo T cell activation in an MHC-unrestricted fashion. CAR-modified T cells can thereby be redirected towards antigens of various structure and composition. Alternatively, T cells can be redirected in an MHC-restricted fashion by using antibody-derived binding domains with TCR-like specificity [20–22]. Genetically engineered with a CAR, modified T cells are amplified ex vivo to numbers suitable for adoptive cell therapy and administered to the patient upon preconditioning. Preclinical and clinical data, which are discussed below, provide strong evidence that peripheral blood T cells from B-CLL patients can successfully be redirected to initiate an effective antitumor response even in advanced stages of the disease.

Success of adoptive therapy with modified T cells, however, depends upon efficient and durable expression of the transgenic CAR. Mostly murine *γ*-retroviral vectors are used to modify T cells taking advantage of its small size, stable transgene integration, and the ability to generate vector batches of high titres. Up to 10^10^ gene-modified T cells can be obtained by retroviral transduction in a Good Manufactoring Procedure-conform manner. However, the strong mitogenic stimulus required for retrovirus transduction may result in T cells which have undergone excessive replication and are suboptimal for an efficient anticancer response. Lentiviral vectors, in contrast, require cytokine prestimulation of recipien T cells which generates T cells with a less differentiated phenotype. Recent developments such as the incorporation of a measles virus envelope protein into viral particles allow transductions with less or without T cell stimulation. Alternatively, naked plasmid DNA or RNA by electroporation is used to obtain CAR-modified T cells; the DNA transfection efficiency is low requiring extensive T cell amplification prior-clinical application. Recent developments in transposon technology suggest that these technologies may also be amenable to clinically modifying T cells in the near future.

## 3. **CD19 Is a Good Target for a Redirected** T Cell** Attack of B-CLL Cells**

The target for CAR-mediated tumor cell recognition is crucial for the therapeutic success, and several issues have to be considered. The target must be expressed on the cell surface of the tumor cell to be recognized by CAR-modified T cells. Most “tumor-associated antigens”, however, are self-antigens and not exclusively expressed on tumor cells but on cells of healthy tissues as well. Malignan T cells moreover show extreme flexibility, loose target antigen expression, and the tumor may recover despite an ongoing immune response. Ideal would therefore be a target molecule which is causally associated with the malignant phenotype since antigen-loss tumor cell variants which are not furthermore recognized by CAR-redirected T cells would loose their malignancy and enter senescence. 

To selectively target B-CLL cells, CD19 seems to be a good target since it fulfils some although not all of the above-cited criteria. CD19 is physiologically expressed on B-lineage cells of almost all stages, from the pro-B-cell to mature B-cell, and is in particular absent from plasma cells, hematopoietic stem cells and other tissues. CD19 decreases the threshold for B-cell activation by assembling with the antigen receptor which enables B-cells to respond to different antigens in a specific and sensitive manner. B-lineage leukemia cells including B-CLL express CD19 at high levels, even during progression of the disease. Targeting CD19 is therefore ideal for redirected therapy of B-CLL, and no myelosuppression, apart from B-cell depletion, or other organ toxicities is expected due to the restricted CD19 expression. CD20 is expressed by nearly the same cells as CD19; targeting CD20 may be an alternative, however, with the same expected side effects. 

The receptor tyrosine kinase-like orphan receptor 1 (ROR1) may be an alternative target for eliminating B-CLL cells [23]. Compared to CD19, ROR1 has the advantage that it is not expressed on normal B-cells. ROR1 is an oncofetal antigen and expressed by undifferentiated embryonic stem cells but not by major adult tissues apart from low levels in adipose tissue and at an early stage of B-cell development. CAR-modified T cells with specificity for ROR1 eliminate B-CLL cells but not mature normal B-cells. The expression on some normal tissues, however, suggests potential toxicity of ROR1-specific T cells.

## 4. The Car Redirected T Cell Antitumor Response Benefits from Costimulation

According to the “two-signal paradigm,” T cells require in addition to the TCR/CD3 signal (“signal 1”) a second signal called costimulation or “signal 2” to sustain pro-longed activation, to improve proliferation, to increase cytokine secretion, and to avoid anergy. CD28 costimulation increases bcl-2 and bcl-xL expression [24] and thereby improves resistance towards activation-induced cell death by preventing apoptosis. To provide CD28 costimulation along with CAR signalling, the CD3*ζ* endodomain was combined with the CD28 costimulatory domain in a so-called “second generation” CAR with combined CD28-CD3*ζ* signalling moiety [25, 26]. There is increasing support for the use of alternative costimulation, for example, via 4-1BB (CD137) or OX40 (CD134), both members of the CD28 family. Each of these costimulatory domains modulates the redirected effector functions in a different fashion including cytokine secretion, proliferation, and prevention from activation-induced cell death [27, 28]. CD28 costimulation mediates IL-2 secretion [26, 29, 30]; without simultaneous costimulation through the native B7-CD28, 4-1BB, and OX40 costimulation do not induce IL-2 although both increasing IFN-*γ* secretion. CD28-CD3*ζ* CAR stimulated T cells thereby indirectly increase antitumor efficacy by sustaining survival, proliferation, and recruiting other activated bystander T cells in the tumor environment. OX40 and 4-1BB costimulation, however, is superior in preventing activation-induced cell death and in sustaining T cell survival. These observations lead to CARs with two costimulatory domains to further improve T cell potency and persistence by augmenting the levels of anti-apoptotic proteins [31]. Combining CD28, OX40 and CD3*ζ* as well as CD28 with 4-1BB and CD3*ζ* induced superior T cell expansion and cytokine secretion. 4-1BB-CD3*ζ* alone, however, is superior in antileukaemia activity in vivo compared to CD28-CD3*ζ* or CD28-OX40-CD3*ζ* CARs [32]. CAR-mediated T cell cytotoxicity as revealed by in vitro short-term assays, however, is independent of costimulation. Taken together combining costimulatory domains with CD3*ζ* allows for specifically modulating T cell effector functions in order to sustain a long-lasting antitumor response.

Costimulation, moreover, provides benefit when T cells enter the immune-suppressive environment of tumors. Immune repression, mostly more pronounced in solid tumors, is mediated infiltrating suppressive cells and by tumor cells itself through repressive cytokines or the altered metabolism which results in the depletion from essential nutrients or the accumulation of immunosuppressive metabolites in the microenvironment. Metabolites with suppressive activity include indolamine-2,3-dioxygenase (IDO), arginase, inducible nitric oxide synthetase (iNOS), and lactate dehydrogenase (LDH)-A, all repressing the adaptive immune response. One of the repressive cytokines is TGF-*β* expressed by a variety of tumor cells on the cell surface and secreted into the tumor environment and expressed by repressive immune cells. CD28 costimulation counteracts repression of T cell proliferation by TGF-*β* thereby improving the antitumor response of redirected T cells [33]. Treg cells infiltrating the tumor mass repress a CAR-redirected T cell antitumor response [34]. Since Treg cells require IL-2 for survival and repression effector, T cells equipped with a CAR which is deficient in inducing CD28-mediated IL-2 secretion exhibit a superior antitumor response in presence of Treg cells [35]. Taken together, appropriate costimulation can, at least partially, counteract tumor-mediated immune repression.

## 5. **In Vitro Evidence for the Efficacy of CAR-Redirected** T Cells** toward B-CLL Cells**

**Figure 1:**
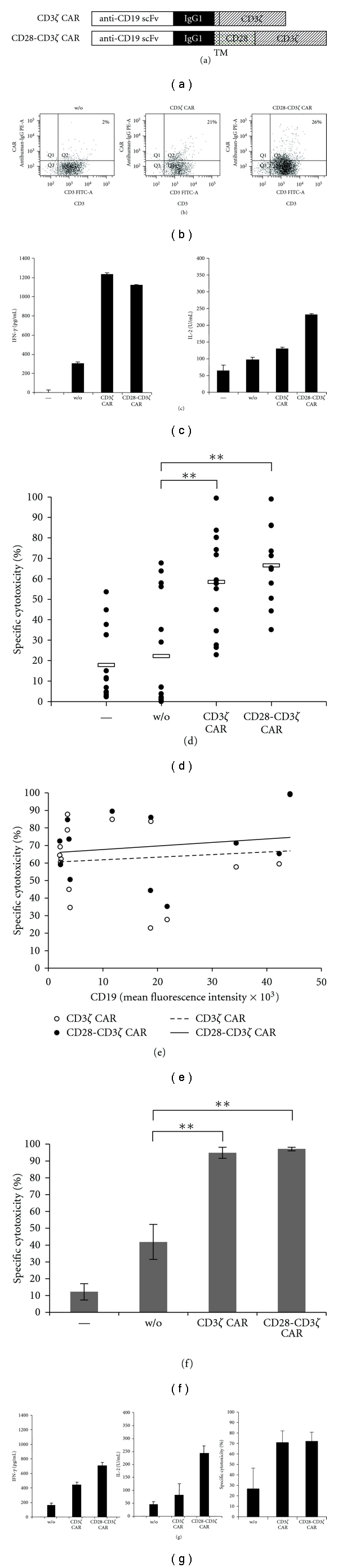
Anti-CD19 CAR redirects engineered T cells towards CD19^+^ B-CLL cells. (a) Schematic diagram depicting the modular composition of the recombinant CD19-specific chimeric antigen receptor (CAR). scFv: single chain fragment of variable region antibody; IgG1: hinge-CH_2_CH_3_ domain of IgG1; TM: transmembrane domain; CD3*ζ*: intracellular domain of CD3*ζ*; CD28: intracellular domain of CD28. (b) Peripheral blood T cells were transduced by retroviral gene transfer to express the respective anti-CD19 CAR. CAR expression was monitored by flow cytometry upon staining with a FITC-conjugated anti-CD3 antibody and a PE-coupled antihuman IgG1 Fc antibody directed against the extracellular IgG1 CAR domain. (c) CAR-mediated T cell activation was monitored by recording IFN-*γ* and IL-2 secretion upon coincubation of anti-CD19 CAR-engineered T cells (5 × 10^5^ cells/well) with primary CD19^+^ B-CLL cells (1 × 10^5^ cells/well). After 24 hrs, IFN-*γ* and IL2 in the coculture supernatant were determined by ELISA. (d) Anti-CD19 CAR-engineered T cells (10^5^ cells/well) from healthy donors were coincubated with B-CLL cells (10^5^ cells/well), and the viability of B-CLL cells was monitored by a flow cytometry-based assay after 24 hrs. B-CLL cells were identified by staining for CD5 and CD19, T cells by staining for CD3, dead cells by staining with 7-AAD. The number of viable B-CLL cells was determined using “Rainbow beads” (Becton Dickinson) as standard. Spontaneous cytolysis is recorded by incubation of B-CLL cells without T cells (−). CAR-redirected cytolysis was calculated in comparison to cytolysis by T cells without CAR (w/o). (e) The efficacy in specific cytolysis by anti-CD19 CAR-engineered T cells (data from D) is independent of the CD19 expression level on B-CLL cells as determined by mean fluorescence intensity of CD19 staining. (f) Anti-CD19 CAR-engineered T cells engineered with anti-CD19 CAR with CD3*ζ* and CD28-CD3*ζ* signalling domain, respectively, were incubated with allogeneic peripheral blood B-cells (purity > 95%) (1 × 10^5^ cells/well each). B-cells alone (−) and B-cells mixed with un-modified T cells without CAR (w/o) were incubated as control. Specific cytotoxicity towards B-cells was recorded after 24 h by a flow cytometry-based assay. T cells were identified by CD3 staining, B-cells by CD5 and CD19 staining, apoptotic cells by 7-AAD staining. (g) CAR-engineered T cells from B-CLL patients lyse autologous B-CLL cells. T cells from B-CLL patients (*n* = 3) were engineered with the CD3*ζ* and CD28-CD3*ζ* CAR, respectively, both with specificity for CD19, and coincubated with autologous CD19^+^ B-CLL cells (each 1 × 10^5^ cells/well) for 24 hrs. Cytokine release into the culture supernatant was determined by ELISA. CAR-engineered patient's T cells showed improved cytotoxicity towards autologous B-CLL cells, indicated by decrease in B-CLL cell viability, compared to nonmodified T cells. Data represent the mean ± standard error of mean. Statistic calculations are based on Student's *t*-test; ∗∗ represents *P* < 0.001.

T-cells engineered with a CD19-specific CAR with CD3*ζ* or combined CD28-CD3*ζ* signalling domain are currently explored for targeting B-CLL cells. Both CARs can efficiently be expressed on peripheral blood T cells and activate T cells in a CD19-dependent fashion indicated by increase in proinflammatory cytokines including IFN-*γ* (Figures 1(a) and 1(b)). CAR-driven T cell activation is antigen-specific since unmodified T cells or T cells with a CAR of irrelevant specificity are not activated upon binding to CD19^+^ cells. In contrast to CD3*ζ* CAR signalling, T cells triggered by the CD28-CD3*ζ* CAR, furthermore, secrete IL-2 (Figure 1(c)). Anti-CD19 CAR T cells from healthy donors exhibit cytolytic activity towards B-CLL cells in vitro (Figure 1(d)). The redirected cytolytic activity in a short term in vitro cytotoxicity assay is not substantially higher by CD28-CD3*ζ* compared to CD3*ζ* CAR T cells which is in accordance to other reports using CARs of different specificities [29]. The efficacy in both the CD3*ζ* and CD28-CD3*ζ* CAR-redirected cytolysis does not furthermore increase with the level of CD19 expression on B-CLL cells (Figure 1(e)) implying that the CD19 levels on B-CLL cells are high enough to cross-link the anti-CD19 CAR for synapse formation and signalling. Anti-CD19 CAR-modified T cells, however, do not distinguish between normal B-cells and B-CLL cells leading to the elimination of normal B-cells as well (Figure 1(f)).

Peripheral blood T cells from B-CLL patients can be redirected towards autologous B-CLL cells. CAR-engineered T cells increase IFN-*γ* secretion when engaging autologous B-CLL and additionally secrete IL-2 when stimulated by the CD28-CD3*ζ* CAR (Figure 1(g)). Patient's T cells efficiently lyse autologous B-CLL cells in a short term in vitro assay. 

**Figure 2:**
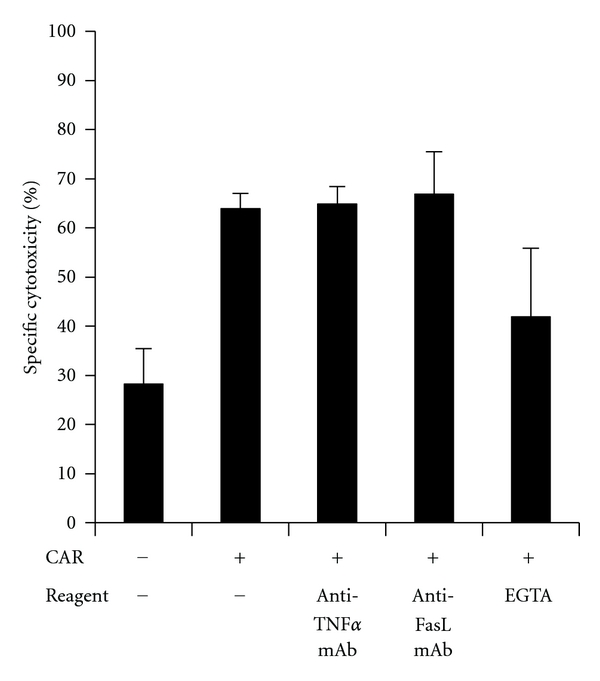
CAR-redirected T cells eliminate B-CLL cells predominantly via granule-mediated cytolysis. Anti-CD19scFv-CD3*ζ* CAR T cells were co-incubated (1 × 10^5^ cells/well) with B-CLL cells (5 × 10^5^ cells/well) in presence of the blocking anti-Fas-ligand antibody (10 *μ*g/mL), the neutralizing anti-TNF*α* antibody (10 *μ*g/mL), and EGTA (2 mM), respectively. Viability of B-CLL cells was monitored by flow cytometry after 18 hrs. As controls, the neutralizing capacities of the anti-TNF-*α* and anti-FasL antibodies were assessed by incubation of sensitive indicator cells with the respective reagents and antibodies in a cytotoxicity assay (data not shown).

B-CLL cells are resistant to Fas-mediated cell death [36] rising the question how CAR-engineered T cells execute lysis of B-CLL cells. Basically, cytolytic T cells can lyse targe T cells by a granzyme/perforin-dependent mechanism, which requires Ca^2+^ release, via Fas/FasL interaction or via TNF-*α*. The cytolytic activity of CD19-specific CAR T cells is blocked by EGTA while nearly unaltered upon blocking Fas and TNF*α* (Figure 2) indicating that cytolysis is predominantly executed by a granule-dependent pathway to overcome Fas resistance of B-CLL cells. 

**Figure 3:**
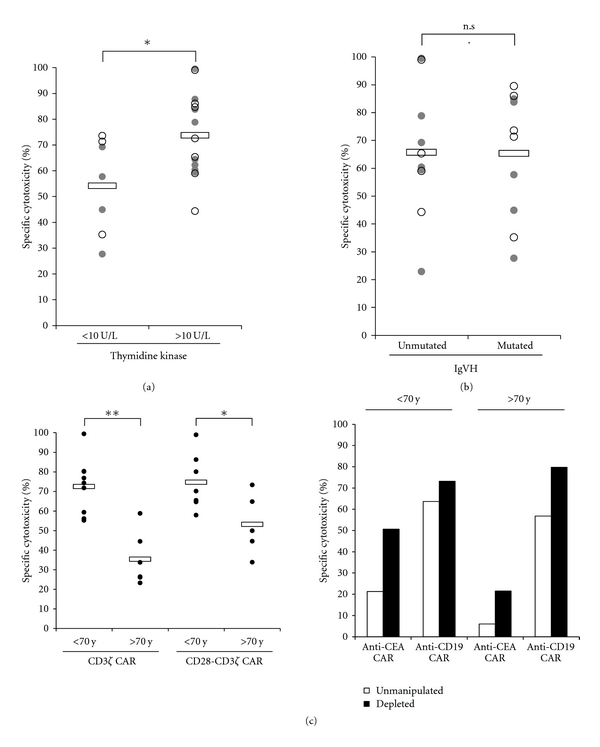
ZAP70-positive B-CLL cells are more efficiently eliminated by CAR-redirected T cells in vitro. The efficacy in specific cytolysis by CAR-redirected T cells (data from Figure 1(d)) was plotted against (a) serum thymidine kinase-1 levels (<10 U/L versus >10 U/L), (b) mutated versus unmutated status of the immunoglobulin heavy chain variable region of B-CLL cells, and (c) patient's age (<70 yrs versus >70 yrs). Closed circles represent CD3*ζ* CAR, open circles CD28-CD3*ζ* CAR-mediated B-CLL killing. Depletion from CD25^high^ Treg cells improves redirected cytolysis of B-CLL cells as exemplarily shown for two patients. Statistic calculations were performed using Student's *t*-test, *P < 0.05; **P < 0.001.

High-serum thymidine kinase-1 levels identify a subgroup of patients with CLL at high risk for disease progression [37]. Thymidine kinase-1 is involved in the salvage pathway for DNA synthesis, found in the cytoplasm of dividing cells and is absent in resting cells [38]. Cycling tumor cells are more susceptible to a redirected T cell attack compared to resting cells. Accordingly, B-CLL cells with high proliferative capacities from patients with high-serum thymidine kinase-1 levels, that is, >10 U/L, are more efficiently eliminated by redirected T cells in vitro than B-CLL cells from patients with low-thymidine kinase-1 levels (Figure 3). Susceptibility to a CAR-redirected T cell attack is not correlated with other clinical prognostic factors like mutation of the immunoglobulin heavy chain variable region (IgVH) locus. B-CLL cells in the population of blood mononuclear cells from patients of younger age are more efficiently eliminated than cells from >70 year patients. This is likely due to the fact that regulatory T (Treg) cells increase in numbers in the blood with progression of the disease and thus with increasing patient's age [39]. Consequently, depletion from Treg cells accordingly increased T cell -mediated elimination of B-CLL cells.

## 6. Murine Models Demonstrate SuccessfulTargeting of CD19^+^ Leukaemia Cells In Vivo

The CAR-redirected T cell response towards CD19^+^ targe T cells was extensively studied in murine models. In most studies, immunodeficient mice were engrafted with primary human CD19^+^ leukaemia cells or cell lines before adoptive transfer of engineered T cells [36, 40, 41]. Other models use murine tumor cells which were equipped with the human target antigen and attacked by engineered murine T cells. Although these studies demonstrated the elimination of malignan T cells from immunodeficient mice, they do not reflect the clinical situation of the immunocompetent patient who is tolerant to CD19 self-antigen and experienced a long adaptation to the growing tumor cell mass. 

A most recently reported model takes these issues into account [18]. Anti-CD19scFv-CD28-CD3*ζ* CAR engineered syngeneic T cells which target murine CD19 were adoptively transferred to immune competent mice which expressed CD19 on healthy B-cells and on a transplanted, syngeneic lymphoma. Along with the antilymphoma activity anti-CD19 CAR-engineered T cells exhibited profound and long-lasting activity against healthy CD19^+^ B-cells without recovery up to 200 days after adoptive T cell transfer. This is in accordance with clinical experience where lymphoma patients treated with anti-CD19 CAR T cells showed lasting and complete depletion of B-cells [42]. In the clinical context, B-cell depletion is manageable and can, at least partially, be alleviated by immunoglobulin replacement.

An alternative model was described by Cheadle et al. [43]. In contrast to the above-described model, T cells were engineered with a CD3*ζ* CAR without CD28 costimulatory domain. CAR-modified T cells showed a profound antilymphoma effect in the syngeneic mouse accompanied by temporary depletion of healthy B-cells. Whether the difference depends on the presence or absence of CD28 costimulation in the context of CAR-mediated T cell activation or on the different CD19-binding domains remains to be explored.

## 7. Lymphodepletion Improves AntitumorEfficacy of Redirected T Cells

The immunocompetent mouse model [18], moreover, indicated the crucial role of preconditioning for antilymphoma efficacy of adoptively transferred T cells. When CAR modified T cells were transferred into mice without prior total body irradiation, only marginal antilymphoma activity was observed with minimal improvement in survival compared to untreated mice. In contrast, all mice survived when irradiated prior to adoptive T cell transfer. These and other data confirm that lymphodepletion before adoptive T cell therapy is crucial for antitumor efficacy [44, 45]. Increasing preconditioning improves antitumor efficacy of adoptive T cell therapy [46]. The lymphodepleting regimen is by itself not sufficient to elicit antitumor responses, their benefit obviously results from the produced environment which favours persistence and expansion of the adoptively transferred T cells. 

Several mechanisms may contribute to the observation [47]. T cell homeostasis in number and function under normopenic conditions in a normal host is tightly controlled by multiple redundant mechanisms to protect the host from uncontrolled immune responses against pathogens and from harmful autoimmunity. Inducing lymphopenia by treatment with cyclophosphamide and fludarabine or by total body irradiation is assumed to provide a selective advantage to adoptively transferred T cells. Nonmyeloablative treatment eliminates regulatory T cells and other repressive cell populations and eliminates immature dendritic cells which anergize T cells. Cell populations competing for the same survival and stimulatory cytokines, like IL-2, IL-7, IL-15, and IL-21, are eliminated as well (“cytokine sinks”) which enhances the availability of those factors to adoptively transferred T cells. Under these conditions of an induced proinflammatory environment adoptively transferred T cells have selective advantage to undergo homeostatic expansion and to improve effector functions. In this context, it is worthwhile to note that the day of adoptive T cell transfer in relation to preconditioning seems to be crucial since T cells given at day 2 after stem cell transplantation show superior amplification and persistence than cells given at later days [48]. Safe nonmyeloablative lymphodepleting preconditioning protocols are developed and are currently used in adoptive T cell trials as summarized in Table 1. 

Experimental data indicate that increased intensity lymphoablation by high-dose total body irradiation given together with haematopoietic stem cell transplantation further improves efficacy of adoptive T cell therapy [50]. With intensified ablation, the levels of pro-inflammatory cytokines increased and tumor treatment efficacy improved. Increased intensity of preconditioning, however, goes against the current trend in hematopoietic stem cell transplantation to reduce treatment-related adverse events by nonmyeloablative strategies. Myeloablation-associated toxicity needs therefore be titrated against the benefit of improved antitumor efficacy.

## 8. **Clinical Trials with CAR-Engineered** T Cells** in the Adoptive Therapy of B-CLL**

A panel of anti-CD19 CARs were characterized by means of laboratory methods during the last decade. Good Manufacturing Processes (GMP) conform procedures are established to modify T cells from the peripheral blood ex vivo with a CD19-specific CAR and to subsequently expand engineered T cells to numbers sufficient for adoptive cell therapy [51]. The processes allow generating clinically relevant doses of CAR-engineered T cells in about 2-3 weeks in a semiclosed culture system. After expansion, the diversity of the TCR repertoire is preserved, and the CD4 : CD8 T cell ratio did not change or rather increased. Modification of T cells by DNA transfection turned out to be less efficient compared to viral transduction [52]. 

CAR-modified T cells are currently explored in a number of phase I trials for the therapy of B-CLL and other B-cell malignancies (Table 1). Patients are receiving CAR-engineered T cells in advanced stages of the disease, and the optimal approach is currently being explored, in particular the optimal dose and the intensity of lymphodepletion [49]. Lessons learnt from pre-clinical animal studies moreover suggest superior antitumor performance of CD28 and 4-1BB costimulatory CARs.

Most severe side effects reported for trials with adoptively transferred CAR T cells were not treatment related; some, although manageable, required temporary discontinuation of therapy and protocol modification. In a phase I trial, however, a treatment-related death of an extensively pre-treated CLL patient occurred shortly after lymphodepletion and infusion of CD28-CD3*ζ* CAR T cells at a total dose of 3 × 10^7^ T cells per kg [53]. The patient was in the second dose escalation cohort. In contrast to patients in the first cohort who received the same number of T cells without developing significant adverse events, this patient was the first to receive cyclophosphamide pretreatment for lymphodepletion. The syndromes patient developed immediately after T cell transfer are consistent with an inflammatory cytokine cascade after lymphodepletion which gave the clinical picture of renal failure and adult respiratory distress syndrome. Although toxicity did not appear to be directly caused by the modified T cells, investigators modified the protocol by reducing T cell dose and administering T cells in split doses to improve safety. Two patients treated on this trial under the modified protocol tolerated treatment without notable toxicities.

There is a clear correlation between persistence of modified T cells and clinical outcome [54]. To improve T cell persistence, Epstein Barr virus- (EBV-) specific T cells are used assumed that those T cells receive optimal and continuous costimulation through their native TCR resulting in longer survival and redirected cytotoxicity-mediated through their CAR. Triggered by chronic EBV infection, CAR-modified EBV-specific T cells, like other virus-specific T cells, may be superior providing a long-lasting antitumor response [54]. A recently initiated trial at Baylor College of Medicine (NCT00608270) aims to address this issue for the treatment of B-cell malignancies. Alternative strategies avoiding the need to isolate EBV-specific T cell clones from each individual patient are needed to facilitate broad application in long term. Application of homeostatic interleukins like IL-7, IL-15, and IL-21 [36, 55, 56] is certainly not selective in expanding modified T cells. 

T cell persistence, moreover, seems to differ when T cell clones or polyclonal T cell populations were transferred. Modified T cells obtained from limiting dilution procedures persisted for 1–3 weeks, compared to 5–9 weeks when patients received T cells from bulk cultures together with low-dose IL-2 for 14 days [57]. In that trial, modified T cells showed indications of efficacy in the treatment of B-cell lymphoma since two treated patients maintained complete partial responses, and four patients exhibited stable disease.

## 9. Challenges for the Targeted Immunotherapy of B-CLL

### 9.1. The CAR Design

The impact of the individual CAR domains on redirected T cell activation was recently discussed in detail [58]; we here focus on particular issues related to anti-CD19 CARs. The CAR-targeting domain has significant impact on redirected T cell activation. The anti-CD19 scFvs currently used are derived from monoclonal antibodies of different affinities targeting different epitopes of CD19. Since both affinity and epitope impact CAR-mediated T cell activation, the optimal combination needs to be identified. 

Most binding domains were derived from murine antibodies. The generation of human antimouse antibody responses was reported in some trials including the generation of anti-idiotypic antibodies which blocked CAR-mediated antigen recognition [59, 60]. An antibody immune response against modified T cells limits the persistence of modified T cells; CARs with humanized domains will therefore be beneficial.

A “spacer” domain between the scFv and the trans-membrane domain improves binding to antigen by overcoming steric hindrance in attaining sufficient proximity to the target antigen. Most CARs therefore harbour the human IgG1 hinge-CH2CH3 region between the scFv and trans-membrane domain. The same spacer, however, may lead to nonspecific activation of effector cells through interaction with Fc receptors which can be, at least partly, prevented by a modified Fc region [61]. Other spacer regions like CD8 can alternatively be used.

While CAR-redirected T cells clearly exhibit antigen-specific and dose-dependent recognition of target cells, engineered T cells sometimes produce small but potentially crucial amounts of pro-inflammatory cytokines, such as IFN-*γ*, even when the targeted antigen is absent [62]. A recent study demonstrates that inactivation of the first and third CD3*ζ* ITAM decreased non-specific IFN-*γ* production by anti-CD19 CAR-modified T cells without impairment of the antileukaemia activity [18]. The Fc*ε*RI *γ* chain which harbours one ITAM in contrast to the three ITAMs in the CD3*ζ* chain may alternatively be used as implied by earlier studies [63]. 

“First generation” CARs transmit the signal through the CD3*ζ* intracellular chain, “second generation” CARs added a costimulatory domain like CD28, 4-1BB or OX40 to improve T cell persistence and activation. While each of these domains differentially modulates individual effector functions [28], the benefit of each costimulation in mounting the antitumor response needs to be determined. This is moreover required for the most recent “third generation” CARs with combined costimulatory domains.

### 9.2. The Effector T Cell Population

Different T cell populations are currently explored for adoptive cell therapy; it is still unresolved which T cell subset shows best therapeutic performance. There is increasing evidence that cytotoxic effector T cells are not a homogenous population but consist of different subsets with individual phenotypes and functional capacities. Resting CD8^+^ T cells in the peripheral blood exist as naïve, central memory, and effector memory T cells. Effector and central memory T cells can be subdivided on the basis of their expression of homing receptors to lymphoid organs. Effector memory T cells develop effector functions more rapidly than central memory T cells, however, secrete lower amounts of IL-2. In mouse models, central memory T cells engraft, survive better, and exhibit superior antitumor activity than effector memory T cells [64]. Data were confirmed by a study of nonhuman primates [65]. Central memory T cells can efficiently be produced ex vivo by CD3 and CD28 stimulation which can be further augmented by IL-7 and IL-15; CD3 stimulation in presence of IL-2 showed less effective [66]. 

Naïve T cells, however, represent the most common CD8^+^ T cell phenotype and thereby the major source of effector cells. Using T cells transgenically or retrovirally, equipped with a tumor-specific TCR, [67] revealed that naïve T cell-derived effector cells are superior for proliferation and cytokine production than effector cells derived from central memory T cells. Longer persistence of those cells may result in superior antitumor efficacy compared to central memory T cells. The procedures for isolating and modifying naïve T cells from cancer patients in a GMP-compliant manner, however, still need to be developed. 

CD4^+^ and CD8^+^ lymphocytes are most frequently transferred since a mixture of those T cell subsets performs better than either T cell subset alone in preclinical models [68]. Bulk T cells, however, contain regulatory T (Treg) cells which repress the antitumor response [34, 35]. Since CD4^+^ T cell depletion eliminates helper CD4^+^ T cells along with Treg cells and depleting CD25^high^ T cells also eliminates proliferating T cells, a more specific strategy in eliminating Treg cells is needed. 

Issues additionally to be addressed in the near future include the particular immune status and the decreasing T cell number in the peripheral blood of patients in advanced stages of the disease. The efficiency in collecting T cells with sufficient functional capacities will be dependent on the clinical situation in which T cells are collected, that is, a patient in remission with minimal residual disease versus a patient with bulk disease. This situation challenges collecting adequate numbers of T cells to be expanded. Apart from that, patients in advanced stages of the disease accumulate a large number of antigen experienced T cells with diminished activation potential due to decreased CD3*ζ* expression and downstream signalling capacities. Engineering with a CD3*ζ* or CD28-CD3*ζ* signalling CAR may overcome some, but not all defects in “burn-out” T cells of progressed tumor patients. We assume that T cells in advanced stages of terminal differentiation may require additional stimuli to execute their effector functions. On the other hand, low T cell counts in patients with advanced disease may limit the overall efficiency in generating engineered T cells with the consequence that multiple rounds of ex vivo amplifications are required to provide clinically effective T cell numbers. While longer ex vivo stimulation provides higher numbers of CAR-modified effector cells, it remains questionable whether their antitumor potency and proliferative capacity conserves with expansion.

Protective immune response seems to be associated with the ability of adoptively transferred T cells to form memory [64]. Conditions which promote protective memory after adoptive T cell transfer need to be established. 

Although cytotoxic T cells are extremely effective eliminating larger haematopoietic tumor mass and of residual tumor cells in preclinical model systems, other effector cells may be envisaged. Beside T cells, NK cells can be effectively redirected by engineering with CARs [69]. Anti-CD19 CAR NK cells, modified by RNA transfer, showed redirected lysis of CLL cells in vitro [70] providing hope for an alternative effector cell population in adoptive therapy.

T-cells from each individual patient need to be modified, amplified, and tested prior to reinfusion. Local production at each clinical institution requires individually approved cell processing facilities and trained personnel to ensure guideline conform production and the uniformity of the cell product. From the regulatory point of view, one or few central facilities may be advantageous which receive cells from the individual patients and produce the cell product. Once tested for safety parameters the cell product can be shipped in a cryopreserved fashion to the clinical site and locally stored until adoptive transfer to the patient.

### 9.3. Toxicity

T-cells are mostly transduced by retro- or lentivirus infection to obtain CAR modified cells with high efficiencies. As far as safety concerns, mutagenesis by insertion of the CAR encoding transgene needs to be addressed. There is so far no reported experimental evidence that retrovirally modified, mature polyclonal T cells produce clonal amplification upon adoptive transfer [71]. Clinically, malignant transformation was not observed in any case of more than 100 patients who were treated so far with gene-modified T cells which is in contrast to the treatment with genetically modified haematopoietic stem cells. Apart from that, the search for a safer vector system using nonintegrating vectors [72], RNA transfer [73], or targeted recombination into safe sites [74] is still ongoing. 

Since CD19 targeting is not tumor specific, CD19^+^ healthy B-cells are eliminated as well. While this situation is expected to be clinically manageable, selectivity for B-CLL cells may be improved by targeting alternative, more unique surface markers or by simultaneous targeting of two different markers. To improve tumor cell selectivity, CAR-redirected T cell killing can, moreover, be combined with the administration of therapeutic antibodies as shown for the anti-CD20 antibody rituximab which sustains the antitumor activity of anti-CD19 CAR T cells in the treatment of non-Hodgkin's lymphoma [75].

Once adoptively transferred, controlling engineered T cell in vivo represents an important option. High-dose steroids showed effective in eliminating engineered T cells in a recent trial [76]; alternative strategies using tagged receptor molecules which can be targeted by depleting antibodies [77] and an inducible caspase-based suicide system [78] showed efficacy in experimental models.

## 10. The Way Ahead

We think it is quite possible that improvements in all of these and potentially of additional aspects are required for success in the T cell therapy of B-CLL in particular and of malignant diseases in general. Cell dose for minimal toxicity and maximal antitumor efficacy may be different for each CAR, for each effector cell population, for each preconditioning regimen, and others. The complexity of adoptive cell therapy challenges standard clinical trial strategies lastingly established in testing drug-based therapies. At least two aspects have to be taken into account.

First, a standard in assay systems to monitor cell therapy-induced immune responses needs to be defined to allow comparison of data sets from different clinical trials. 

Second, trials differ in such a large number of parameters that it will be problematic to identify those aspects which are critical for the effectiveness or ineffectiveness of a protocol. Table 1 partly illustrates the issue for trials using CD19 CAR-modified T cells. To unambiguously identify the effects of defined changes in clinical trial protocols it will require systematic “one-parameter trials” on the basis of a standard format, in particular with respect to conditions for cell modification, a CAR format, the target, and for preconditioning. The currently recruiting trials using anti-CD19 CAR modified T cells give chance for standardizing and rapidly optimizing the strategy with respect to the discussed parameters [49]. Although toxicities occurred in early-phase trials and caution is still warranted, the potential benefits of adoptive cell therapy with redirected T cells for the therapy of B-cell malignancies should not be abandoned.

## Figures and Tables

**Table 1:** Phase 1 clinical trials using anti-CD19 CAR modified T cells for the treatment of B-cell malignancies (updated and adapted from [49]).

Disease	CAR configuration	Preconditioning	Status of trial	Clinical trials.gov identifier	Clinical trial centre
B-CLL	scFv-CD28-CD3*ζ*	none versus cyclophosphamide	recruiting	NCT00466531	Memorial Sloan-Kettering Cancer Center
B-ALL	scFv-CD28-CD3*ζ*	none	recruiting	NCT00709033	Baylor College of Medicine
B-NHL, B-CLL	scFv-CD28-CD3*ζ* versus scFv-CD3*ζ*	none	recruiting	NCT00586391	Baylor College of Medicine
B-NHL, B-CLL	scFv-CD28-CD3*ζ* versus EBV/scFv-CD3*ζ*	none	recruiting	NCT00608270	Baylor College of Medicine
B-ALL	scFv-CD28-CD3*ζ*	cyclophosphamide	recruiting	NCT01044069	Memorial Sloan-Kettering Cancer Center
B-lymphoma, B-CLL	scFv-CD28-CD3*ζ*	fludarabine plus cyclophosphamide	recruiting	NCT00924326	National Cancer Institute
B-lymphoma/leukemia	scFv-41BB-CD3*ζ* versus scFv-CD3*ζ*	variable		NCT00891215	The University of Pennsylvania
B-NHL	scFv-CD28-CD3*ζ*	BEAM-R		NCT00968760	MD Anderson Cancer Center
refractory B-cell lymphoma/leukemia	scFv-CD3*ζ*	fludarabine plus low dose cyclophosphamide	recruiting		The University of Manchester, UK
